# A narrative review of antibody–drug conjugates in *EGFR*-mutated non-small cell lung cancer

**DOI:** 10.3389/fonc.2023.1252652

**Published:** 2023-12-01

**Authors:** Robert Hsu, David J. Benjamin

**Affiliations:** ^1^ Department of Internal Medicine, Division of Medical Oncology, Norris Comprehensive Cancer Center and Hospital, University of Southern California, Los Angeles, CA, United States; ^2^ Hoag Family Cancer Institute, Newport Beach, CA, United States

**Keywords:** non-small cell lung cancer (NSCLC), epidermal growth factor receptor (*EGFR*), antibody drug conjugate (ADC), tyrosine kinase inhibitor resistance (TKI resistance), targeted therapy resistance

## Abstract

In the past 15 years, non-small cell lung cancer (NSCLC) treatment has changed with the discovery of mutations and the development of new targeted therapies and immune checkpoint inhibitors. Epidermal growth factor receptor (*EGFR*) was the first mutation in NSCLC to have a drug that was FDA-approved in 2013. Osimertinib, a third-generation tyrosine kinase inhibitor, is approved as first-line therapy for advanced NSCLC and in the adjuvant setting for Stage IB-IIIA resected NSCLC. However, resistance to osimertinib is inevitably an issue, and thus patterns of resistance to *EGFR*-mutated NSCLC have been studied, including *MET* amplification, *EGFR* C797X-acquired mutation, human epidermal growth factor 2 (HER2) amplification, and transformation to small cell and squamous cell lung cancer. Current management for *EGFR*-mutated NSCLC upon progression of *EGFR* TKI is limited at this time to chemotherapy and radiation therapy, sometimes in combination with the continuation of osimertinib. Antibody–drug conjugates (ADCs) are made up of a monoclonal antibody linked to a cytotoxic drug and are an increasingly popular class of drug being studied in NSCLC. Trastuzumab deruxtecan has received accelerated FDA approval in HER2-mutated NSCLC. ADCs offer a possible solution to finding a new treatment that could bypass the intracellular resistance mechanism. In this review article, we summarize the mechanism of ADCs and investigational ADCs for *EGFR*-mutated NSCLC, which include targets to MET amplification, HER3, Trop2, and EGFR, along with other ADC targets being investigated in NSCLC, and discuss future directions that may arise with ADCs in *EGFR*-mutated NSCLC.

## 1 Introduction

The development of targeted therapies has changed the systemic treatment landscape of non-small cell lung cancer (NSCLC) (1–9). Erlotinib was the first targeted therapy FDA-approved for epidermal growth factor receptor (*EGFR*) mutations in 2013 (10). Osimertinib, a third-generation tyrosine kinase inhibitor (TKI), is approved as first-line therapy for advanced NSCLC as well as in the adjuvant setting for stage IB-IIIA-resected NSCLC (1, 11). However, osimertinib and other TKIs develop resistance patterns (12, 13). The most common patterns of resistance in *EGFR*-mutated NSCLC treated with osimertinib frontline are *MET* amplification, *EGFR* C797X-acquired mutation, and human epidermal growth factor 2 (HER2) amplification. Up to 15% of *EGFR*-mutated NSCLC treated with osimertinib transforms into small cell lung cancer (SCLC) or squamous cell carcinoma (12, 13).

*EGFR*-mutated NSCLC with progression of disease treatment options on osimertinib is limited, as oligoprogression or CNS metastasis is often treated with radiation therapy while continuing osimertinib (14, 15). Those with visceral metastasis beyond oligoprogression or SCLC transformation are treated with chemotherapy (14, 15). Immune checkpoint inhibitors (ICIs) have little benefit and significant toxicity in *EGFR*-mutated NSCLC (16, 17). The IMMUNOTARGET registry showed that ICI by itself in *EGFR*-mutated NSCLC had a shorter median progression-free survival (PFS) of 2.1 months and a lower overall response rate (ORR) of 12% compared to other targetable mutations (18). Attempts to combine *EGFR* TKIs with ICIs were not successful, as the TATTON study, which involved an *EGFR* TKI and durvalumab, was discontinued due to toxicity primarily due to interstitial lung disease (ILD) (19). Other combination ICI and *EGFR* TKI studies (KEYNOTE-021 and Checkmate 012) showed no OS benefit with severe grade 3+ hepatotoxicity in five of seven patients receiving gefitinib in KEYNOTE-021 (19, 20). Furthermore, the use of ICI followed by osimertinib led to severe immune-related adverse events in 15% of patients, particularly within 3 months of osimertinib use (17). KEYNOTE-789, a phase 3 randomized controlled trial of chemotherapy and pembrolizumab in TKI-resistant *EGFR*-mutated patients, showed no PFS or OS benefit (21). The lack of success of ICIs in *EGFR*-mutated NSCLC has limited treatment options upon progression of first-line *EGFR* TKI.

Antibody–drug conjugates (ADCs) consist of a monoclonal antibody attached to a cytotoxic drug (22, 23). These drugs have highly specific targeting abilities and can effectively kill the targeted cells and theoretically avoid toxicity toward other nontargeted cells (22, 23). Gemtuzumab ozogamicin was the first ADC given approval for treatment of patients >60 years with CD33^+^ acute myeloid leukemia who are not candidates for aggressive therapy (24). In solid tumors, one of the first ADC targets was HER2, a transmembrane protein in the erb-b2 receptor tyrosine kinase 2 (*ERBB2*). HER2 protein overexpression is observed in about 20% of NSCLC patients (25). Trastuzumab deruxtecan (T-DXd), an ADC-targeting HER2, was the first ADC in NSCLC given FDA-accelerated approval in 2022 (8, 26).

The development of T-DXd has led to more antigens being targeted in NSCLC for ADCs. ADCs could circumvent the *EGFR* cell signaling pathway and provide new treatment options for *EGFR*-mutated patients who progress on osimertinib. We will discuss ADC structure, mechanism of action, and targets currently in development in patients with *EGFR*-mutated NSCLC.

## 2 ADC structure and mechanism of action

ADC consists of a monoclonal antibody, typically immunoglobulin G, bound to the target antigen of cells in the tumor. The ADC then fuses with the lysosome, leading to cytotoxic “payload” release that causes death of the targeted cancer cell (22, 23). When choosing an antigen, antigens chosen to be targets for designed ADCs are antigens that have much greater expression in cancer cells than in noncancer cells. For example, *ERBB2*, one of the early targets for ADC development, is expressed 100-fold in tumor tissues more than in nontumor tissues (22, 23, 27). The antibody and payload are connected by the linker and help with ADC stability. Cleavable linkers break down and release the payload based on either a specific pH or a specific lysosomal protease, while noncleavable linkers release the payload following internalization of ADCs by lysosome or proteases (22, 23, 28). The payload is where the ADC acts on the cancer cell and induces cell death. The payloads include agents that affect microtubule stability, inhibit topoisomerase 1, or cleave the DNA (22, 23, 29). There is a bystander effect where the drug, after internalization and degradation, is released across the cell membrane to kill adjacent cells (22, 23, 30). Finally, the drug-to-antibody ratio (DAR) is an important property of ADCs. DAR is the average number of drug molecules (payload) conjugated to the antibody. To calculate DAR, newer ADCs utilize high-performance liquid chromatography-ultraviolet spectroscopy, which adjusts for changes to the antibody or heavy/light chain hydrophobicity due to conjugated payloads, though other approaches include liquid chromatography-quadruple time-of-flight mass spectrometry and UV-Vis (31). Earlier ADCs developed had a DAR of 2–4, but newer ADCs such as trastuzumab deruxtecan have a DAR of 8 (31).

## 3 ADC targets for *EGFR*-mutated NSCLC

### 3.1 *MET* amplification

**Figure 1:**
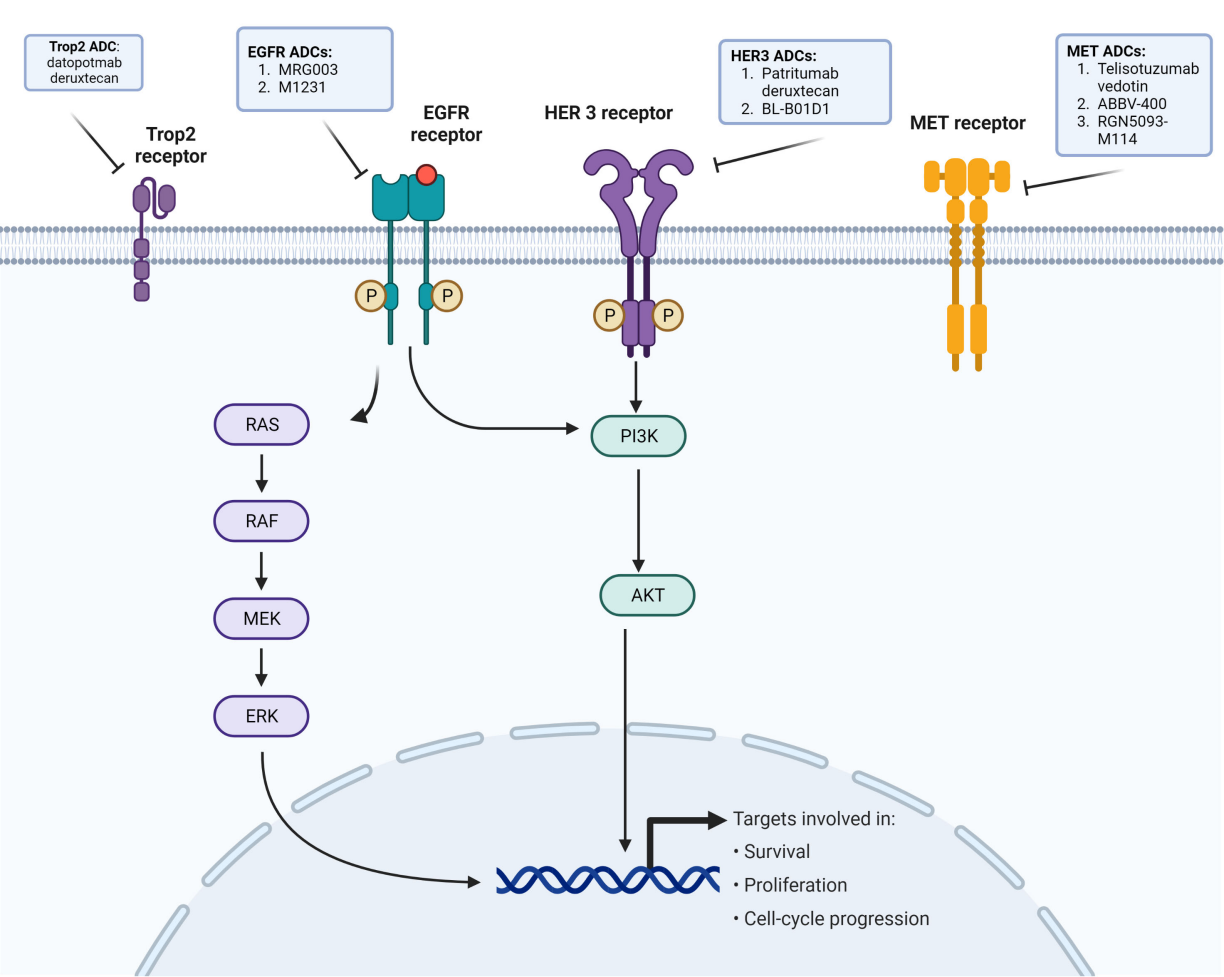
EGFR cellular pathway and purpose, and receptors with ADCs in development against *EGFR*-mutated NSCLC.

*MET* amplification is a common resistance mechanism in *EGFR*-mutated NSCLC, and so ADCs targeting *MET*-dysregulated NSCLC are being developed to address this (12, 13) (**Figure 1**). Amivantamab is a bispecific monoclonal antibody targeting *MET* and *EGFR*. In the CHRYALSIS study in which amivantamab is given with lazertinib, a third-generation *EGFR* TKI, the ORR was 36% (95% CI, 23–51) with 39% having a duration of response (DOR) longer than 6 months. Of those patients with disease progression after osimertinib and chemotherapy, the ORR was 29% (95% CI, 17–42) and the median DOR was 8.6 months (95% CI, 4.2–not reached). The most common grade ≥3 treatment-related adverse events was infusion-related reaction (7%) (NCT04077463) (32). The MARIPOSA-2 trial comparing amivantamab–chemotherapy, amivantamab–chemotherapy–lazertinib, and chemotherapy only showed significantly increased PFS results in amivantamab–chemotherapy and amivantamab–lazertinib–chemotherapy versus chemotherapy (median PFS of 8.2 and 8.3, respectively, versus 4.2 months), but with decreased hematologic adverse events in amivantamab–chemotherapy arm versus amivantamab–lazertinib–chemotherapy (NCT04988295) (33). An ADC involving MET dysregulation is telisotuzumab vedotin (Teliso-V), which is an ADC consisting of a c-Met antibody (ABT-700) and a microtubule inhibitor (monomethyl auristatin E) (34). In the LUMINOSITY phase 2 trial involving c-MET overexpressing NSCLC, there was a subcohort of 43 patients in which the ORR was 11% (95% CI, 3.9–25.1). Peripheral sensory neuropathy was the most common adverse event (25.0%), with two grade 5 adverse events possibly related to Teliso-V (sudden death and pneumonitis in one patient each) (NCT03539536) (34). Other ADCs being developed that may be promising in the future for MET-amplified *EGFR*-mutated NSCLC include ABBV-400, which targets c-MET and topoisomerase, in which there is an ongoing phase 1 study (NCT05029882) along with a biparatropic MET × MET ADC REGN-M114 (NCT04982224) (35, 36). Preclinical activity of REGN-M114 showed activity in *EGFR* mutant NSCLC with *PTEN* loss or *MET* Y1230C mutation cell lines that were pretreated with osimertinib and savolitinib (37). There is currently a phase 1 study (NCT04982224) looking at REGN-5093-M114 in *MET*-overexpressing advanced solid tumors (**Table 1**).

**Table 1:** Current/key trials of ADCs in *EGFR*-mutated NSCLC.

Drug	Target	Payload	Trial	ORR	DOR
MET amplification					
Telisotuzumab vedotin	c-MET	Vedotin (monomethyl auristatin)	LUMINOSITY (NCT03539536) (*n* = 43 *EGFR* mutant nonsquamous cell) (34)	11.6 (3.9–25.1) in *EGFR* mutants	NR (3.0, NR)
ABBV-400	c-MET	Topoisomerase	NCT05029882 (36)		
REGN5093-M114	MET	M114 (M24, maytansine derivative)	NCT04982224 (35)		
AZD9592	c-MET and EGFR	Topoisomerase I	NCT05647122 (40)		
HER3					
Patritumab deruxtecan	HER3	Deruxtecan (DXd)	NCT03260491(*n* = 57 *EGFR* mutant receiving HER3-DXd 5.6 mg/kg) (44)	39.0 (26.0–52.4)	6.9 (3.1–NE)
			HERTHENA-Lung01 (NCT04619004) (*n* = 225) (45, 46)	29.8 (23.9–36.2)	6.4 (4.9–7.8)
			HERTHENALung02 (NCT05338970)		
			NCT04676477 (combination treatment of HER3-DXd with osimertinib)		
BL-B01D1	HER3	Topoisomerase I	NCT05194982 (*n* = 34 *EGFR* mutant) (47)	61.8 (43.6–77.8)	
EGFR-directed ADCs					
MRG003	EGFR	Monomethyl auristatin E	NCT04838548		
M1231	EGFR/MUC1	Hemiasterlin-related microtubule inhibitor	NCT04695847		
Trop2					
Datopotamab deruxtecan	Trop2	Topoisomerase I	TROPION-PanTumor01 (NCT03401385) (*n* = 34 NSCLC with actionable genomic alterations including 29 *EGFR* mutant) (56)	35.0 (19.7–53.5)	9.5 (3.3–NE)
			TROPION-Lung05 (NCT04484142) (*n* = 49, including 56.9% with *EGFR* mutations) (58)	35.8 (27.8–44.4)	7.0

AZD9592 is a bispecific ADC targeting *EGFR* and cMET with a monovalent bispecific IgG platform with an increased affinity toward c-MET to reduce *EGFR*-related toxicities, along with a topoisomerase I payload (38). AZD9592 monotherapy *in vivo* in patient-derived xenograft models showed a 30% reduction of tumor in 41% of *EGFR* mutant NSCLC tumors at 2 mg/kg and a 73% response rate in those treated at 8 mg/kg (38, 39). Currently, there is a phase 1 trial on AZD9592 monotherapy in combination with osimertinib in *EGFR* mutant NSCLC (NCT05647122) (40) (**Table 1**).

### 3.2 HER3

There remains a large percentage of *EGFR*-mutated NSCLC patients receiving *EGFR* TKI with no known identifiable resistance mechanisms (12, 13). *ERBB3* (HER3) is found in 83% of primary NSCLC tumors and is also expressed in other solid tumors (41). HER3 is found in a greater proportion of *EGFR* mutant NSCLC compared to *EGFR* wild-type NSCLC (42). Patritumab deruxtecan (HER3-DXd) is a HER3-directed ADC composed of a monoclonal antibody to HER3 covalently linked to a topoisomerase I inhibitor payload with a DAR ratio of 8 and is cell membrane-permeable, thus allowing cell death both at the target and surrounding tumor cells (43) (**Figure 1**). In a phase 1 study looking at HER3-DXd in metastatic *EGFR*-mutated NSCLC with prior *EGFR* TKI therapy, 57 patients received the dose-recommended HER3-DXd of 5.6 mg/kg q3 weeks (44). The ORR was 39% (95% CI, 26.0–62.4) with a median PFS of 8.2 (95% CI, 4.4–8.3) (44). The ORR in 23 of the 57 patients with known *EGFR*-related resistance mechanisms, excluding T790M, was 35%. HER3-DXd was well tolerated with a low discontinuation rate (9%, 7/81) (44). Treatment-related ILDs in this study occurred in 5% of patients and resolved in all patients after drug discontinuation (44) (**Table 1**).

HERTHENA-Lung01, a phase II study of HER3-DXd in *EGFR*-mutated NSCLC patients with disease progression after *EGFR* TKI and platinum-baseed chemotherapy, demonstrated a promising ORR of 29.8% (95% CI, 23.9–36.2) with a CNS ORR of 33.3% (95% CI, 17.3–52.8) (NCT04619004) (45, 46). Meanwhile, there is a phase III study comparing HER3-DXd to platinum-based chemotherapy in patients with disease progression after *EGFR* TKI therapy(HERTHENA-Lung02; NCT05338970). Another phase I study involving HER3-DXd in combination with osimertinib in patients who have progressed with osimertinib monotherapy (NCT04676477) is currently recruiting (**Table 1**).

Meanwhile, BL-B01D1 is a first-in-class EGFR × HER3 bispecific ADC linked to topoisomerase I inhibitor via a cleavable linker. Phase I data from a first-in-human study showed an ORR of 61% (95% CI, 43.6–77.8) and a disease control rate (DCR) of 91.2% (76.3–98.1) in heavily treated NSCLC *EGFR*-mutated cases (*n* = 34). All *EGFR*-mutated patients had previous *EGFR* TKI exposure, and 88% of these patients had prior third-generation *EGFR* TKI (NCT05194982) (47). There was an ORR of 40% (25.6–56.7) in NSCLC *EGFR* wild-type patients, and the most common grade 3 or higher treatment-related adverse events were leukopenia (30%), neutropenia (34%), and anemia (15%). No ILD was observed (47) (**Table 1**).

Izalontamab (SI-B001) is another first-in-class novel EGFR × HER3 bispecific antibody. A phase II study looking at SI-B001 plus docetaxel in patients who had failed on anti-PD-1/L1 antibody plus platinum-based chemotherapy had an ORR of 31.3% with no drug-related deaths, suggesting that this agent has activity with a manageable safety profile warranting further investigation (NCT05020457) (48).

### 3.3 EGFR

There have been other ADCs directly targeting *EGFR*, with mixed results. MRG003 is an anti-EGFR humanized immunoglobulin G1 monoclonal antibody conjugated with monomethyl auristatin E via a valine–citrulline linker (**Figure 1**). In a phase 1 study of primarily head and neck squamous cell carcinoma, nasopharyngeal carcinoma, and colorectal cancer patients, the ORR was 40%, 44%, and 0%, respectively (NCT04868344) (49). Currently, a phase II study looking at the efficacy and safety of MRG003 is ongoing in *EGFR*-positive advanced non-small cell lung cancer (NCT04838548) (**Table 1**).

Depatuxizumab mafodotin (ABT-414), which consists of an *EGFR*-specific humanized antibody, a non-cleavable malemidocaproyl linker, and monomethyl auristatin F, was tested for patients with *EGFR*-amplified newly diagnosed glioblastoma but discontinued due to a lack of survival benefit (50). AVID-100, which is another *EGFR*-targeted ADC failed in phase I/II trial in solid tumors due to a lack of efficacy (NCT03094169) (51). M1231 is another ADC that consists of a bispecific antibody targeting Mucin 1 (MUC1) and *EGFR* with a hemiasterlin-related payload; a current phase I trial in solid tumors is ongoing (NCT04695847) (52) (**Figure 1**).

AFM24 is a tetravalent bispecific innate cell engager targeting *EGFR* on tumor cells and CD16A on natural killer cells to enhance antitumor antibody-dependent cellular cytotoxicity (53). A phase 1/2 monotherapy evaluating AFM24 in 14 heavily treated *EGFR* mutant NSCLC patients showed a DCR of 50% and a median duration of therapy of 6.7 weeks (1.0–26.1). There was acceptable safety with one incidence of grade 5 pneumonitis (NCT04259450) (54).

### 3.4 Trop2

Another promising ADC target is trophoblast cell surface antigen (Trop2), which is a transmembrane glycoprotein calcium signal transducer and is overexpressed in over 60% of adenocarcinomas and 75% of squamous cell carcinoma (NSCLC). Datopotamab deruxtecan (Dato-DXd) consists of a Trop2-directed monoclonal antibody conjugated to a topoisomerase I inhibitor via a stable tetrapeptide-based cleavable linker (55) (**Figure 1**). The TROPION-PanTumor 01 trial included 159 NSCLC mostly pretreated patients and had an ORR of 21%–25% (23% at 4 mg/kg, 21% at 6 mg/kg, and 25% at 8 mg/kg) with a median PFS of 4.3–8.2 months (55). In those with advanced/metastatic NSCLC with actionable mutations (*n* = 34), which included 29 *EGFR*-mutated patients, the ORR was 35% (19.7–53.5) with a median DOR of 9.5 months (95% CI, 3.3–NE) (56). The TROPION-Lung05 is looking at patients with actionable genomic alterations previously treated with at least one targeted therapy and one platinum-based chemotherapy, and initial results show ORR of 35.8% (95% CI, 27.8–44.4) and a median DOR of 7.0 months (NCT04484142) (57, 58). Another ADC-targeting Trop2 is sacituzumab govitecan (SG). In the phase I/II IMMU-132 basket trial, there were 54 patients who received 8–12 mg/kg, and the ORR was 16.7% (95% CI, 7.9–29.3), with a median PFS of 4.4 months and a median OS of 7.3 months. About 60% had grade 3 or greater treatment-related adverse events, with neutropenia (42.4%) being most common (59) (**Table 1**).

### 3.5 Additional ADC targets in NSCLC

In advanced NSCLC targeting HER2, ado-trastuzumab emtansine (TDM-1) had an ORR of 44%. However, the median duration of response (DOR) was 3.5 months with a median PFS of 2.8 months and a median OS of 8.1, and thus no further phase III NSCLC trials were pursued due to the limited efficacy (60, 61). T-DXd had an ORR of 55% in advanced HER2-mutant NSCLC with a median PFS of 8.2 months (95% CI, 6.0–11.9 months) and a median OS of 17.8 months (95% CI, 13.8–22.1 months). Drug-related ILD did occur in 26% of patients (8). Consequently, a phase II trial comparing T-DXd 5.4 mg/kg versus 6.4 mg/kg dosing showed similar efficacy in both doses, with a decrease in the adjudicated drug-related ILD rate in the 5.4-mg/kg dose (NCT04644237) (62). In regard to other ADC targets, carcinoembryonic antigen-related cell adhesion molecule 5 (CEACAM5) is highly expressed in about 25% of lung cancers, and a novel ADC targeting this is tusamitamab ravtansine, comprising a humanized monoclonal antibody and a cytotoxic maytansinoid, DM4. Phase 1 data of tusamitamab ravtansine showed ORR 20.3% (95% CI, 12.3–31.7) in high expression of CEACAM5 with grade 3 or greater TEAE in 48% of patients (63). Finally, B7-H3 protein is seen in 80% of NSCLC cases with high protein levels associated with poor prognosis and could be a considered a target for ADC development (64).

## 4 Future directions

While ADCs provide promise, there are multiple considerations. The first consideration is resistance patterns and treatment upon progression from an ADC. Some mechanisms of ADC resistance include decreased antigen expression after exposure to ADC, as seen in some subjects who had received TDM-1; processing of ADC that may lead to less uptake in the cancer cell; and resistance to the payload (65, 66). As ADCs have an increased risk for ILD toxicity, there may be a risk for pneumonitis if patients were to go back to an *EGFR* TKI afterward (67). This is important as newer generation of TKIs are being developed, such as BLU-945 to address *EGFR* C797X resistance, and questions arise as to sequencing between drugs (68, 69).

Another consideration is weighing drug efficacy versus drug toxicity. The incidence of ILD in the key trials has been about 20%, and there are other notable toxicities that can significantly alter the quality of life, such as 20% ocular toxicity seen in TROPION-Lung02 (44, 70). Subsequent analysis of patient-reported outcomes will be vital in determining the benefit of drug efficacy versus drug toxicity.

CNS response rate is an important consideration. *EGFR*-mutated NSCLC leads to brain metastasis in about 50%–60% of patients, so finding an agent with CNS activity is vital. Earlier ADCs did not have good blood–brain barrier penetration due to a lack of homogeneity of the ADC along with a suboptimal drug-to-antibody ratio (71). The phase 1 HER3-DXd study in NSCLC patients has an ORR of 36.4% in patients with brain metastasis and a median DOR of 7.3 months, suggesting some activity, but more work needs to be done, particularly given the high risk of CNS metastasis in these patients (72).

In addition, up to 15% of *EGFR*-mutated patients have the risk of small-cell lung cancer transformation on progression (12, 13). There have not been any reported trials on ADCs in these patients, but delta-like ligand 3 (DLL-3) is a target expressed in 80% of SCLCs. Rovalpituzumab tesirine was an ADC in development in SCLC that was discontinued due to lack of benefit, but bispecific T-cell engagers such as tarlatamab have shown promising responses in heavily treated SCLC (73, 74). However, the mechanism of SCLC and squamous cell transformation in these patients needs to be understood so that we can focus on a specific target for this population.

Finally, combinations with ADCs are a consideration. Many trials combine chemotherapy, but with *EGFR*-mutated NSCLC, it will be interesting to see if a combination of ADC with *EGFR* TKI as studied in HER3-DXd with osimertinib will prove to be more efficacious and not more toxic than ADC monotherapy, and if so, if this combination will be more effective than ADC with chemotherapy (NCT04676477).

## 5 Conclusion

There has been tremendous progress in *EGFR*-mutated NSCLC since the approval of erlotinib in 2013, but with that come challenges, notably acquired resistance and treatment upon disease progression (10). ADCs are an increasingly studied drug class with more antigen targets being found. With trastuzumab deruxtecan approved for HER2-mutated NSCLC, there are promising developments in other ADCs targeting *HER3*, *MET* amplification, and *EGFR* directly. Future challenges involve the combination of ADCs with other drugs, sequencing of drugs, toxicity, particularly ILD incidence, and controlling brain metastasis in patients with CNS involvement.

## Author contributions

RH designed the review and wrote the original manuscript draft. DB produced **Figure 1**, reviewed, and edited the manuscript. All authors contributed to the article and approved the submitted version.
